# Green Synthesis of Selenium Nanoparticles by Cyanobacterium *Spirulina platensis* (abdf2224): Cultivation Condition Quality Controls

**DOI:** 10.1155/2021/6635297

**Published:** 2021-05-29

**Authors:** Shohreh Alipour, Sara Kalari, Mohammad Hossein Morowvat, Zahra Sabahi, Ali Dehshahri

**Affiliations:** ^1^Pharmaceutical Sciences Research Center, Shiraz University of Medical Sciences, Shiraz, Iran; ^2^Department of Quality Control, School of Pharmacy, Shiraz University of Medical Sciences, P.O. Box 71468-64685, Shiraz, Iran; ^3^Department of Pharmaceutical Biotechnology, School of Pharmacy, Shiraz University of Medical Sciences, Shiraz, Iran; ^4^Medicinal Plants Processing Research Center, Shiraz University of Medical Sciences, Shiraz, Iran

## Abstract

Selenium nanoparticles (SeNPs) are well-known bioactive compounds. Various chemical and biological methods have been applied to SeNP synthesis. *Spirulina platensis* is a widely used blue-green microalgae in various industries. In this study, the biosynthesis of SeNPs using sodium selenite and *Spirulina platens* has been developed. The SeNP synthesis was performed at different cultivation condition including pH and illumination schedule variation. The SeNPs were characterized by FT-IR, XRD, size, and zeta potential measurements, and the antioxidant activities of selected SeNPs were evaluated by DPPH and FRAP assays. FT-IR analysis showed the production of SeNPs. The 12 h dark/12 h light cycles and continuous light exposure at pH 5 led to the production of stable SeNPs with sizes of 145 ± 6 and 171 ± 13 nm, respectively. Antioxidant activity of selected SeNPs was higher than sodium selenite. It seems that green synthesis is a safe method to produce SeNPs as well as a convenient method to scale-up this production.

## 1. Introduction

Recently, great attention has been directed to selenium as one of the significant dietary supplements. The importance of selenium in human diet comes from the fact that this element is present in the structure of proteins including glutathione peroxidase, thioredoxin reductase, and deiodinases. These enzymes act as scavengers of reactive oxygen spices (ROS) with antioxidant activity [1, 2]. As oxidative stress and ROS play critical roles in several pathological conditions such as cancer, sufficient selenium may act as an effective player in the normal cell proliferation as well as homeostatic mechanisms [2, 3]. Organic and inorganic selenium, including selenite, selenomethionine, and Se-methyl selenocysteine, has been administered commonly in animals and humans with selenium deficiency. Some of the selenium supplements, particularly the inorganic forms, have shown toxicity in higher nutritional doses. Therefore, SeNPs were developed to reduce its toxicity and improve its biological activities [4]. Nowadays, researchers are seeking for the synthesis and production of SeNPs due to their unique properties. Several reports indicated that the red color nanosized form of selenium showed improved *in vivo* bioavailability and less toxicity compared with the micro or macro forms [5, 6]. Also, the nanoforms of selenium have higher biodegradability and biocompatibility than silver or gold nanoparticles. Interestingly, considerable anticancer and antioxidant activities have been reported for selenium nanoparticles. There are various suggested mechanisms for the anticancer effects of SeNPs such as cell cycle arrest and apoptosis [7, 8]. Also, selenium nanoparticles have been used for wound dressing purposes [9]. Taken together, SeNPs have shown great potential to be used in biomedical applications as well as food supplements.

Several chemical and physical methods have been suggested to prepare SeNPs. In chemical methods, metallic precursors and reducing agents are employed to synthesize SeNPs [8, 10]. Sodium selenite, selenium dioxide, sodium selenosulfate, and selenious acid are different metallic precursors in the chemical synthesis of SeNPs [8, 11]. Environmental toxicity and instability of the nanoparticles are disadvantages of the chemical synthesis of these particles [8, 10]. The particle stability could be improved by adding stabilizers or decorators to the preparation media such as chitosan, glucose, and poly(ethylene glycol) [1]. To reduce the hazardous effects of chemical synthesis, the physical approach (top-down approach) is employed to reduce the size of particles through microwave irradiation or ultrasonication [3]. Although these methods may lead to the preparation of uniformed nanoparticles, the high cost and sophisticated instruments particularly in terms of industrial production may limit their wide applications. Therefore, there is a growing attention on the application of green synthesis of SeNPs in order to reduce the hazardous environmental effect of chemical synthesis and high expenses of physical methods [12]. Biological methods could be used as one of the modest approaches to prepare nanoparticles at ambient temperature [10, 13]. These reactions could be carried out without metal catalyst at nonharsh conditions. This ecofriendly method could be performed by plant extracts as well as various bacteria. There are several reports on the production of SeNPs by *Withania somnifera* leave extract [14], *Emblica officinalis* fruit extract [15], fenugreek extract [16], *Vitis vinifera* (raisin) extract, *Sulfurospirillum barnesii*, *Bacillus selenitireducens*, *Selenihalanaerobacter shriftii*, and *Zooglea ramigera* [17–19]. The biological synthesis of SeNPs by these methods resulted in the production of uniform nanoparticles with significant stability. Among various biological methods suggested for the green preparation of SeNPs, there are limited reports on the application of cyanobacteria as photosynthetic prokaryotes for the production of selenium nanoparticles. These organisms have been widely investigated for agricultural and ecological applications including biofertilizers [13]. Also, their efficiency for biotransformation of various pharmacologically active molecules such as steroids has been reported. The potential of these organisms to produce a wide variety of primary and secondary metabolites makes them as proper candidates for the reduction of metal ions into the nanoparticle form. There are several investigations on the potential of cyanobacteria particularly *Spirulina platensis* in the production of various metal nanoparticles such as silver and gold [20] and tellurium [21]. *Spirulina platensis* is blue-green algae with medical and nutritional properties which is commercially available as human functional food [21, 22]. In the present study, we investigated the ability of *Arthrospira (Spirulina) platensis* (*S*. *platensis)* for the production of SeNPs. The investigation was focused on the evaluation of different cultivation medium including pH and illumination time on the size and polydispersity of the SeNPs. The optimized SeNPs were then tested for their antioxidant activity by FRAP and DPPH tests.

## 2. Methods and Materials

### 2.1. Chemicals and Reagents

In order to prepare suitable media, all chemicals and solvents were purchased in analytical grade from Sigma Chemical Co. (St. Louis, USA). Sodium selenite (Na_2_SeO_3_) was purchased from Merck Chemicals Co. (Darmstadt, Germany).

### 2.2. Cultivation of *S. platensis*

*S. platensis* (abdf2224) was obtained from Algae Bank of Iran (Shiraz, Iran) in Zarrouk's media. Ten milliliters of the cyanobacterial samples were diluted in 90 mL of medium and incubated at 25°C with constant shaking. The samples were illuminated continuously with light with 1400 ± 200 lux intensity.

### 2.3. *S. platensis* Growth Pattern

In order to evaluate the growth pattern of the *S. platensis*, during 10 days, at determined time points, samples were withdrawn from *S. platensis* culture and the biomass was harvested and centrifuged at 11 × 10^3^ rpm for 10 min. The cyanobacterial biomass was washed and dried, and the weight of the dried biomass was considered as the growth index of the cyanobacteria. The weight of cyanobacteria was calculated in triplicate. The maximum specific growth rate (*μ*_max_) was determined using the following equation (Equation (1)), and the data was expressed in day^−1^:

(1) $$\begin{array}{c} \mu_{\max}\frac{\ln X_{2}-\ln X_{1}}{t_{2}-t_{1}}, \end{array}$$

where *X*_1_ and *X*_2_ are the dried cell weight (DCW, g mL^−1^) and (*t*_2_ − *t*_1_) represents the time for increasing cell concentration from *X*_1_ to *X*_2_ in the exponential phase.

### 2.4. SeNP Green Synthesis by *S. platensis*

*S. platensis* was cultured in the suitable medium, and the sodium selenite aqueous solutions (ranging from 2 to 8 mM) were added to the cyanobacterial samples. The media were incubated at room temperature under continuous lightening with constant shaking. Sodium selenite (2 mM) without cyanobacteria was maintained at the same condition as negative control.

The change in the solution color was considered as the indicator for the synthesis of selenium nanoparticles. To obtain a quantitative production pattern for SeNPS, the samples of culture media within 10 days were centrifuged and scanned using a UV-spectrophotometer in the range of 200-700 nm.

Following the synthesis of selenium nanoparticles, the color of media changed from green to orange and finally into red. After completion of the synthesis reaction, the samples were centrifuged at 15 × 10^3^ rpm for 20 min. The unreacted materials were removed by washing with double distilled water followed by the centrifugation at the same condition. Finally, the nanoparticles were freeze-dried.

### 2.5. Cultivation Condition Optimization

The effect of medium pH and illumination time on the particle size and polydispersity index (PDI) of produced SeNPs was evaluated; hence, the cyanobacterial samples were treated at different conditions as follows.

#### 2.5.1. Growth Medium pH

In order to evaluate the effect of medium pH on the production of SeNPs, the cyanobacterial samples were prepared at the same condition as described above and treated with sodium selenite (4 mM). The pH of the media was adjusted at 5, 6, 7, and 8, and the samples were incubated at room temperature under continuous lightening with constant shaking.

#### 2.5.2. Growth Medium Illumination

In order to evaluate the effect of lightening time on the production of SeNPs, the cyanobacterial samples were prepared at optimized pH and treated with optimum sodium selenite concentration. The samples were incubated at room temperature with constant shaking. Various patterns of lightening were used including 24 h dark/24 h light, 16 h dark/8 h light, 12 h dark/12 h light, and 8 h dark/16 h light. The last sample was incubated at continuous lightening condition (Table 1).

### 2.6. Characterization of SeNPs

#### 2.6.1. Size, Distribution, and Zeta Potential

The measurement of the size, polydispersity index (PDI), and zeta potential of the samples was carried out by the Nanotrac Flex In-Situ Particle Size Analyzer (Microtrac, Germany) at 25°C. All samples were analyzed in triplicate.

#### 2.6.2. SEM-EDX Analysis

Scanning electron microscopy–energy-dispersive X-ray (SEM-EDX) observation of SeNPs was performed using a TESCAN MIRA3 instrument (Czech Republic) operating at 0-15 KeV. Samples were filtered and dried before measurements. The material was gold coated using Ion Sputter Coater Hitachi E1010. Energy-dispersive X-ray (EDX) analysis was carried out by the same instrument and employed to know the elemental compositions of the particles. Images were acquired at 50 and 100 kV magnification.

#### 2.6.3. Fourier Transform Infrared Spectroscopy (FT-IR) Analysis

To study the presence of functional groups on SeNP surface, FT-IR analysis was carried out. For FT-IR analysis, the dried powders were grinded with KBr pellets and analyzed on a Vertex70 (Bruker, Germany) FT-IR spectrometer instrument at a resolution of 4 cm^−1^ and the FT-IR spectra were attained at 400–4,000 cm^−1^ wave numbers against potassium bromide background. The peaks obtained were plotted as % transmittance in *X* axis and wave number (cm^−1^) in *Y* axis.

#### 2.6.4. X-Ray Diffraction (XRD) Analysis

The XRD patterns of sodium selenite and the optimum SeNP sample were obtained by XRD-D8 ADVANCE (Bruker, Germany) using Cu Ka 1.5406 A° radiation. The measurement was recorded over the range of 10–70 (2ϴ).

#### 2.6.5. DPPH Assay

In this study, radical scavenging ability was evaluated using DPPH **(**1,1-diphenyl-2-picryl-hydrazyl) assay. To perform the assay, 200 *μ*L of DPPH (100 mM) and 20 *μ*L of SeNP dispersion were mixed in well of microplate and the microplates were incubated at 25°C for 30 min in dark. The absorbance was measured at 495 nm using a microplate reader [23].

#### 2.6.6. FRAP Assay

To determine the antioxidant activity of SeNPs, FRAP (Ferric Reducing Antioxidant Power) assay was performed. Briefly, TPTZ (2,4,6-tripyridyl-S-triazine) solution (10 mmol/L) in HCl (40 mmol/L), FeCl_3_ (20 mmol/L), and acetate buffer (0.3 mol/L, pH 3.6) were used in this assay. Acetate buffer, FeCl_3_, and TPTZ were mixed before use. The mixture was then heated to 37°C. Twenty *μ*L of each sample and 180 *μ*L of FRAP reagent were mixed in a 96-well microplate reader (37°C for 10 minutes). The absorbance of the complex was measured at 593 nm [24].

### 2.7. Statistical Analysis

Data are presented as the mean ± SD. The differences were examined by the one-way ANOVA test. *p* value ≤ 0.05 was considered statistically significant.

## 3. Results

### 3.1. *S. platensis* Growth Pattern

**Figure 1:**
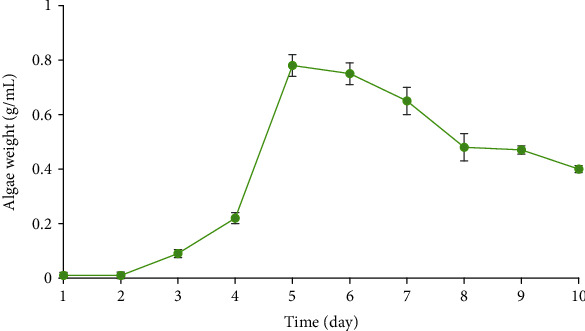
Growth curve of *Spirulina platensis* (abdf2224) (*n* = 3).

*S. platensis* (abdf2224) was cultured in Zarrouk's media. The results of the growth curve of the cyanobacterial sample have been shown in Figure 1. The *S. platensis* (abdf2224) growth pattern exhibited a sigmoidal growth curve with a 2-day lag phase which led to a log phase that was started on the 3^rd^ day after culturing and reached to a maximum on the 5^th^ day. The decline phase started on the 6^th^ day. The *μ*_max_ was determined to be 0.547 day^−1^ at the exponential phase.

### 3.2. SeNP Green Synthesis by *S. platensis*

**Figure 2:**
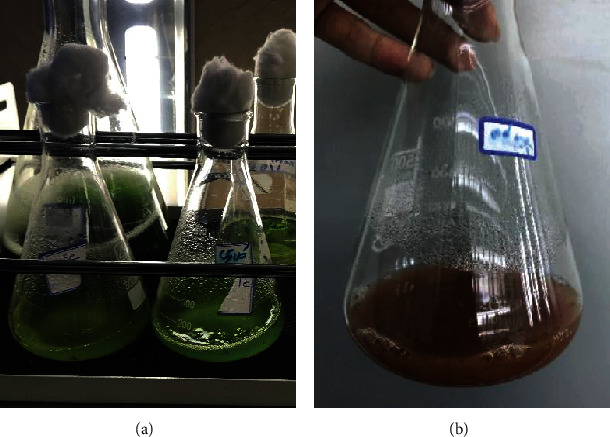
Color change from green to red following the addition of sodium selenite (8 mM) to the cyanobacterial cultures: (a) day 1 and (b) day 6.

**Figure 3:**
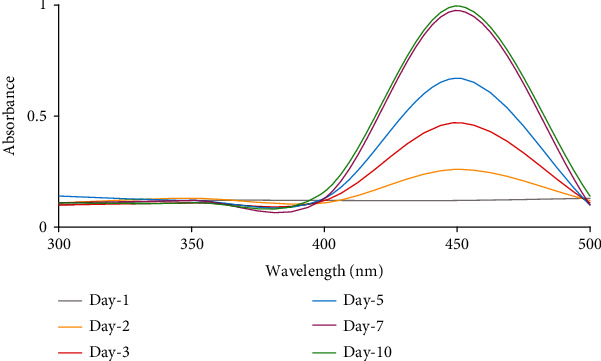
UV-Vis absorption spectra of SeNPs during 7 days of incubation.

The precursor (i.e., sodium selenite) was added at four various concentrations ranging from 2 to 8 mM. The results revealed that no color change was observed for the samples at the concentrations of 2, 4, and 6 mM. However, the samples containing sodium selenite (8 mM) showed a significant change in the medium color from green to orange and red indicating the formation of SeNPs (Figure 2). The quantitative production pattern for SeNPs indicated that UV absorbance in 400-500 nm was increased within 7 days, and after the 7^th^ day, there were no significant differences in absorbance (Figure 3).

### 3.3. Cultivation Condition Optimization

#### 3.3.1. Growth Medium pH

**Figure 4:**
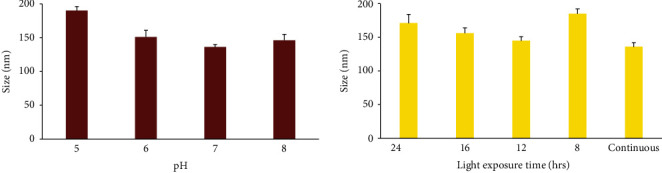
The effect of various pH of medium and light exposure time frames on the SeNP size produced by *Spirulina platensis* (abdf2224).

**Figure 5:**
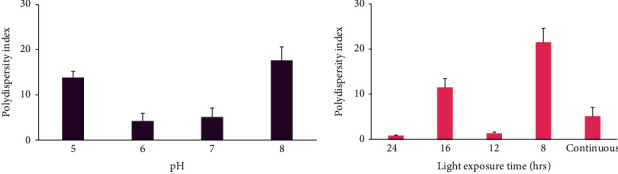
The effect of various pH of medium and light exposure time frames on the SeNP PDI produced by *Spirulina platensis* (abdf2224).

The effect of medium pH on particle size and PDI of synthesized SeNPs is reported in Figures 4 and 5. The particle sizes were in the range of 136-190 nm which indicated that nanoparticle size was similar at pH 6, 7, and 8 (*p* > 0.05) while at pH 5, SeNP size was significantly larger (*p* < 0.05). The PDI was in the range of 4.2-17.6 and showed significantly smaller values in pH 6 and 7 than pH 5 and 8 (*p* < 0.05).

#### 3.3.2. Growth Medium Illumination

The effect of various illumination cycles on SeNP size and PDI was evaluated at optimized pH condition. As it was demonstrated in Figures 4 and 5, smaller nanoparticles were formed in continuous illumination condition (*p* < 0.05) while the PDI values were substantially lower at 24 h dark/24 h light and 12 h dark/12 h light condition (*p* < 0.05).

### 3.4. Characterization of SeNPs

#### 3.4.1. Size, Distribution, and Zeta Potential

In order to evaluate the effect of pH on the particle size, PDI, and zeta potential of SeNPs, the measurement was performed at optimized pH condition at different light exposure as described above (Figures 4 and 5, Table 1). The synthesized SeNPs with the lowest size and PDI were used for further evaluations. The SeNP size was in the range of 136-190 nm. The PDI was in the range of 0.84-21.5, and the zeta potential was negative (-50.5-65.8 mV).

#### 3.4.2. SEM-EDX Analysis

**Figure 6:**
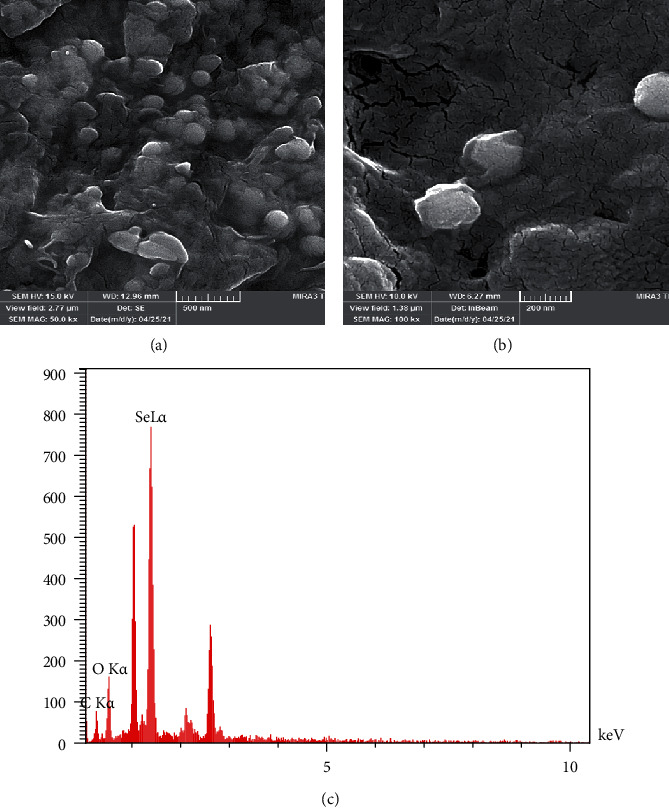
(a) SEM, 50 kV, (b) SEM, 100 kV, and (c) EDX analysis of SeNPs-1 produced by *Spirulina platensis* (abdf2224).

Scanning electron microscopic image (SEM) of the selected SeNPs and EDX signal analysis are presented in Figure 6. According to the SEM image (Figures 6(a) and 6(b)), the nanoparticles were in spherical shape with the particle size of around 100 nm. The elemental analysis showed selenium signal along with carbon and oxygen group peaks. The result confirmed the presence of selenium with 44.99% (wt.) in the sample. Also, carbon (29.9%) and oxygen (25.1%) signals were detected in the analysis.

#### 3.4.3. FT-IR Analysis

**Figure 7:**
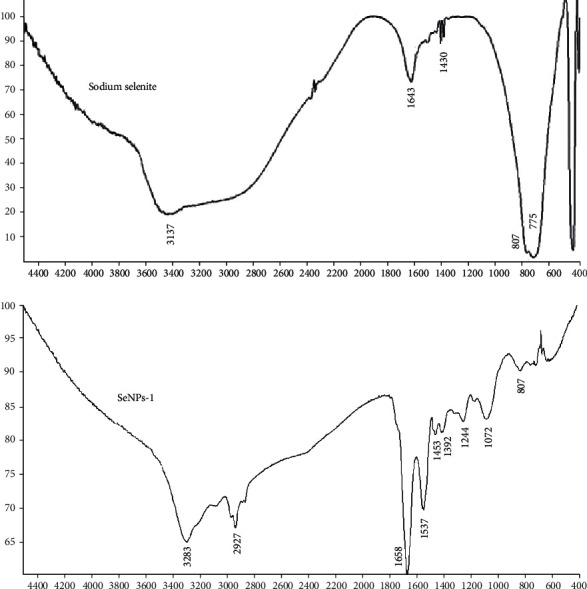
FT-IR spectrum of sodium selenite and SeNPs-1.

The results of sodium selenite and selected synthesized SeNP FT-IR analysis are shown in Figure 7. Sodium selenite peaks were typically presented at 725, 807, and 1430 cm^−1^ while in SeNPs, the mentioned peak intensity was reduced indicating the formation of SeNPs. According to the previous results, intense peaks at 3265, 2927, 1642, 1396, and 1072 cm^−1^ correspond to *Spirulina.*

#### 3.4.4. XRD Evaluation

**Figure 8:**
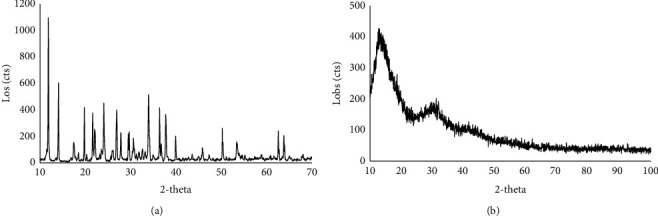
XRD pattern of sodium selenite (a) and SeNPs-1 (b).

The XRD pattern of sodium selenite and the selected synthesized SeNPs are presented in Figure 8. The sharp peaks of sodium selenite at 12, 14.2, 17.5, 19.8, 21.7, 22.3, 24.2, 26.8, 28, 29.5, 30.8, 34, 36.5, 37.8, 40, 45.8, 50.5, 53.5, 62.5, and 63.8 2*θ* indicating a crystalline structure which were not present in SeNPs-1 XRD pattern. Thus, SeNP amorphous structure (Figure 8(b)) may demonstrate that the crystalline structure of sodium selenite (Figure 8(a)) was converted to SeNPs.

#### 3.4.5. Free Radical Scavenging Activity

**Figure 9:**
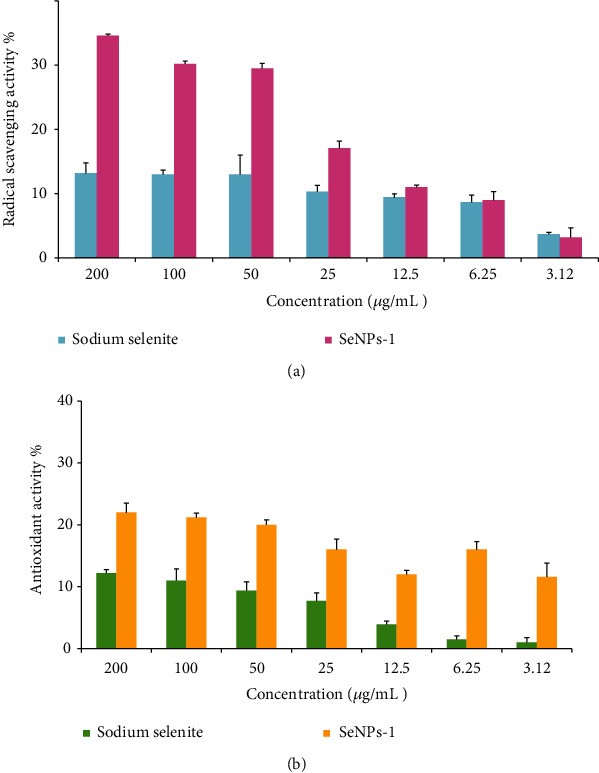
(a) Radical scavenging and (b) antioxidant activity of SeNP-1 and sodium selenite using DPPH and FRAP assay.

The evaluation of antioxidant activity of SeNPs was compared with the parent sodium selenite using DPPH assay. The sodium selenite and SeNPs-1 prepared at pH 7 with various illumination schedules (12 h dark/12 h and continuous light exposure) were tested for potential antioxidant activity (Figure 9). Radical scavenging activity of SeNPs-1 at the highest concentration (200 *μ*g/mL) was 35% while it decreased to 3% at the lowest tested concentration (3.12 *μ*g/mL). The radical scavenging activity of the nanoparticles was 1.1 to 2 times higher than the parent sodium selenite at different concentration range.

#### 3.4.6. Antioxidant Assay

The results of FRAP assay on the optimized SeNPs and sodium selenite are reported in Figure 9. Antioxidant activity of SeNPs-1 at the highest and lowest (200-3.12 *μ*g/mL) concentrations was 22 and 12%, respectively, which was 2 to 12 times higher than antioxidant activity of sodium selenite at different concentration range. Interestingly, the antioxidant activity for SeNPs was substantially higher than sodium selenite at lower concentrations.

## 4. Discussion

### 4.1. *S. platensis* Growth Pattern

The results revealed that after 3 days of incubation, the cyanobacterial samples start the exponential growth which continues for 2 additional days. In other words, the logarithmic phase of growth ends at the fifth day following the inoculation. The cyanobacteria enter the dead phase after day 6. This result could be used as an index to identify the most appropriate time to add the precursor for the synthesis of nanoparticles. In some experiments, the addition of the precursor has been suggested to be done at the exponential phase of growth. However, there are some reports suggesting the addition of such materials at the beginning of the inoculation [25]. The results of our study demonstrated that the cyanobacterial samples enter the dead phase after 6 days and could not be suggested for the synthesis of nanoparticles.

### 4.2. SeNP Green Synthesis by *S. platensis*

The synthesis of SeNPs was detected by the formation of red color in the media. The reduction of Se (IV) to Se (0) results in the medium color change which could be used as a visual indicator for the formation of Se (0) [26–28]. The color change was not detected in the cultures without cyanobacteria confirming the role of *S. platensis* in the reduction of sodium selenite to SeNPs. Since the medium color change was used as a preliminary visual indicator for the formation of nanoparticles, the first changes in the color were observed three days after the addition of sodium selenite. The change of medium color continued for three more days and stopped at the sixth day following the start of the reaction. The results revealed that the reduction of selenite to SeNPs occurs at the logarithmic phase of growth. By increasing the enzymatic ability of the cyanobacteria, the reduction reaction occurs. Therefore, maintaining the cyanobacterial samples in the exponential phase of growth could be considered as a strategy for large-scale production of SeNPs with high yield. The pattern of nanoparticle formation was also monitored by UV-visible spectroscopy. The results of this analysis were consistent with the observation of medium color change as the indicator for nanoparticle formation. The increase in absorbance at 450 nm is associated with the nanoparticle formation which was not observed at days 1 and 2 while the sharp increase occurred at the third day confirming the formation of nanoparticles (Figure 3).

### 4.3. Cultivation Condition Optimization

#### 4.3.1. Growth Medium pH

In order to assess the most appropriate pH for the bioconversion of sodium selenite to selenium nanoparticles, the cyanobacterial cultures were prepared at various pH. At pH of 5, 8, and 10, the color of a medium changed from sea green to pale green indicating the death of cyanobacteria. On the other hand, the cyanobacterial cultures at pH of 6 and 7 demonstrated the medium color change from green to orange and red confirming the formation of selenium nanoparticles. The results of pH optimization revealed that the most appropriate pH for the synthesis of SeNPs using *S. platensis* is around 7. Therefore, the scale-up of the nanoparticle production could be carried out at neutral pH facilitating the application of nonharsh condition for large-scale production.

### 4.4. Characterization of SeNPs

#### 4.4.1. Particle Size, PDI, and Zeta Potential

In order to evaluate the effect of pH on the size of SeNPs, the particle size and PDI measurement was performed at various pH conditions as described above (Figure 4 and Table 1). The largest particle size was achieved at pH 5 where the SeNPs were formed with the particle size of 185 ± 4 nm. On the other hand, the size of nanoparticles at pH 7 was 136 ± 6 nm indicating the formation of the smallest nanoparticles at various pH conditions (*p* < 0.05).

The result of the size measurement at various light exposure time frames (Figure 4 and Table 1) indicated that the 12 h dark/12 h light cycle led to the formation of SeNPs with the size of around 150 nm which was similar to the continuous light exposure condition. However, the other illumination cycles resulted in the formation of larger nanoparticles with the sizes smaller than 200 nm. In other words, various conditions led to the formation of nanoparticles not larger than the critical size range of 200 nm. Interestingly, the polydispersity index for the nanoparticles at pH 7 and the illumination cycle of 12 h dark/12 h light was the lowest value among all the samples (*p* < 0.05). This result demonstrated that the light exposure time and pH of a medium are crucial factors in the formation of homogeneous nanoparticles.

The PDI of SeNPs was significantly lower at pH 6 and 7 (Figure 5 and Table 1). The PDI of SeNPs is presented in Figure 5 and Table 1 indicating the lowest PDI for SeNPs-1 (*p* < 0.05). Finally, the zeta potentials of the SeNPs were also measured as an indicator for the colloidal stability. The results demonstrated that except for SeNPs-3 (pH, 7 with the 12 h dark/12 h light cycle) which have shown the zeta potential around -50.4 (±0.51) mV, the zeta potential for the rest of SeNPs was around -60 mV which resulted in the formation of more stable SeNPs. Previous studies mentioned that the zeta potential range for stable nanoparticles was higher than -30 mV [29]. Considering previous results, SeNPs-1 was selected as the optimized nanoparticles.

#### 4.4.2. SEM-EDX Analysis

The EDX profile shows selenium signal along with carbon and oxygen group peaks. The result indicated that 44.99% (wt.) of the sample had the presence of SeNPs. The detection of carbon and oxygen as impurities may be related to the presence of remained *Spirulina platensis* (abdf2224) which was not fully removed after purification due to its spiral structure. In addition, SeNP oxidation in air before the sample analysis may be the cause of oxygen detection as sample impurity [30].

#### 4.4.3. FT-IR Analysis

FT-IR technique may confirm the presence of various reducing and stabilizing functional groups of metabolites to detect their possible role in the fabrication of SeNPs. Measuring the vibrational frequencies of chemical bonds by FT-IR allows to determine which functional groups exist in the surface of SeNPs [15]. Considering previous studies, several major intense peaks were reported for spirulina around 3265, 2927, 1642, 1396, and 1072 cm^−1^ [31, 32] in which stretching vibration of aliphatic C–H, C–O–H, and *α*-D-glucose are at 2927 and 1072 cm^−1^ which indicated SeNP conjugation to *Spirulina* polysaccharide [32]. The results of FT-IR analysis showed a broad peak at 3283 cm^−1^ that corresponds to O–H stretch alcohols and phenols suggesting a strong hydrogen bonding interaction between selenium and the O-H groups. The absorption peak at 2927 cm^−1^ could be associated with C–H stretch alkynes. The peaks at 1658 and 1453 cm^−1^ correspond to carbonyl groups and amide bonds, respectively, whereas the peak at 1392 cm^−1^ attributed to the C–H bending in alkanes. The typical polysaccharide vibration region (1210–1012 cm^−1^) was shifted in SeNPs to higher frequencies at 1072 and 1244 cm^−1^.

Sodium selenite strong peak at 725 and 807 cm^−1^ exhibited the symmetric and asymmetric Se-O stretching vibration. In the SeNP spectrum, wide single weaker peaks between 1061, 1176, 1240, and 1323 cm^−1^ suggest that the Se–O concentration is much smaller compared to that in pure sodium selenite [33, 34]. These results are consistent with the previous investigations demonstrating the presence of several various functional groups. The various peaks confirm the role of different phytochemicals in facilitating the biosynthesis of SeNPs by reduction and increase the stabilization of nanoparticles. However, the exact structure and identity of such molecules need more detailed investigations [14, 15, 35, 36].

#### 4.4.4. XRD Evaluation

Similar to a previous study [37], the XRD pattern of sodium selenite showed the sharp peaks at 12, 14.2, 17.5, 19.8, 21.7, 22.3, 24.2, 26.8, 28, 29.5, 30.8, 34, 36.5, 37.8, 40, 45.8, 50.5, 53.5, 62.5, and 63.8 2*θ* indicating a crystalline structure while the synthesized SeNP XRD broad peaks (Figure 8) demonstrated the amorphous nature of the nanoparticles. The result of XRD analysis of sodium selenite and SeNPs was consistent with the other investigations in which the crystalline structure of sodium selenite converts to the amorphous SeNPs using green synthesis methods [14, 15, 32, 37–39].

### 4.5. Determination of Antioxidant Activity

#### 4.5.1. DPPH Assay

The antioxidant (radical scavenging) activity of SeNPs was compared with the parent sodium selenite using DPPH assay. The sodium selenite and SeNPs-1 prepared at pH 7 with various illumination schedules (24 h dark/24 h light exposure) were tested for antioxidant activity. The results revealed that the radical scavenging activity of SeNPs-1 was 1.1-2 times higher than sodium selenite (*p* < 0.05) (Figure 9). The higher activity could be associated with the homogeneity of the SeNPs prepared at the definite condition. Oxidative stress results in the significant cellular damages and cellular dysfunction. Therefore, the SeNPs prepared at such condition could show radical scavenging activity [40]. The results obtained in this study are consistent with several investigations reporting the antioxidant activity of selenium nanoparticles [25, 36, 41].

#### 4.5.2. FRAP Assay

In the FRAP assay, the antioxidant material is able to reduce the ferric-tripyridyl triazine (Fe^3+^-TPTZ) complex to blue ferrous (Fe^2+^-TPTZ) complex. In the FRAP method, the antioxidant activity of sodium selenite and SeNPs-1 prepared at pH 7 with various illumination schedules (12 h dark/12 h light exposure) were compared. The results revealed that the antioxidant activity at the highest concentration of SeNPs-1 (200 *μ*g/mL) was 2 times higher than sodium selenite while it was 10-12 times higher than sodium selenite at the lowest concentration (3.12 *μ*g/mL) (*p* < 0.05) (Figure 9). It seems that the homogeneity of the SeNPs is related to higher antioxidant activity. Therefore, the SeNPs prepared at such condition could show higher reducing activity.

## 5. Conclusion

Cyanobacteria have shown several capabilities for the bioconversion or biotransformation of different compounds like steroids. The ability of these photosynthetic organisms to produce nanoparticles has been studied in some investigations. Due to the significant positive effects of selenium in human health, the ability of cyanobacterium *S. platensis* (abdf2224) for the conversion of sodium selenite to SeNPs was investigated in this study. The results revealed that the best condition for the preparation of homogenous nanoparticles was achieved with the light time exposure of 12 h dark/12 h, at pH 7. The amorphous SeNPs were prepared after 3 days of incubation which was associated with the cyanobacterial logarithmic growth phase. The prepared nanoparticles showed significant antioxidant activity compared with the parent sodium selenite suggesting their effectiveness for further studies towards the large-scale production of SeNPs as a supplement.

## Figures and Tables

**Table 1:** SeNP characterization at growth media illumination variation in optimized pH.

	Light exposure time (h)	Darkness time (h)	Size (nm)	PDI	Zeta potential (mv)
SeNPs-1	24	24	171 ± 13	0.84 ± 0.12	−59.36 ± 0.55
SeNPs-2	16	8	156 ± 8	11.5 ± 2	−65.5 ± 1.45
SeNPs-3	12	12	145 ± 6	1.3 ± 0.3	−50.4 ± 0.51
SeNPs-4	8	16	185 ± 7	21.5 ± 3.1	−65.8 ± 1.76
SeNPs-5	Continuous	—	136 ± 6	5.1 ± 0.2	−60.1 ± 0.1

## Data Availability

The data used to support the findings of this study are included within the article.

## References

[1] Sakr T. M., Korany M., Katti K. V. (2018). Selenium nanomaterials in biomedicine--an overview of new opportunities in nanomedicine of selenium. *Journal of Drug Delivery Science and Technology*.

[2] Khurana A., Tekula S., Saifi M. A., Venkatesh P., Godugu C. (2019). Therapeutic applications of selenium nanoparticles. *Biomedicine & Pharmacotherapy*.

[3] Chhabria S., Desai K. (2016). Selenium nanoparticles and their applications. *Encyclopedia of Nanoscience and Nanotechnology*.

[4] Bai K., Hong B., He J., Hong Z., Tan R. (2017). Preparation and antioxidant properties of selenium nanoparticles-loaded chitosan microspheres. *International Journal of Nanomedicine*.

[5] Chaudhary S., Umar A., Mehta S. K. (2014). Surface functionalized selenium nanoparticles for biomedical applications. *Journal of Biomedical Nanotechnology*.

[6] Shanmugam R., Veena P., Santhiyaa R. V., Prasad R., Jha A., Prasad K. Synthesis and characterization of selenium nanoparticles using natural resources and its applications. *Exploring the Realms of Nature for Nanosynthesis*.

[7] Huang Y., He L., Liu W. (2013). Selective cellular uptake and induction of apoptosis of cancer-targeted selenium nanoparticles. *Biomaterials*.

[8] Hosnedlova B., Kepinska M., Skalickova S. (2018). Nano-selenium and its nanomedicine applications: a critical review. *International Journal of Nanomedicine*.

[9] Ahmed M. K., Moydeen A. M., Ismail A. M., el-Naggar M. E., Menazea A. A., el-Newehy M. H. (2021). Wound dressing properties of functionalized environmentally biopolymer loaded with selenium nanoparticles. *Journal of Molecular Structure*.

[10] Menon S., KS S. D., R S., S R., S V. K. (2018). Selenium nanoparticles: a potent chemotherapeutic agent and an elucidation of its mechanism. *Colloids and Surfaces B: Biointerfaces*.

[11] Xia Y., You P., Xu F., Liu J., Xing F. (2015). Novel functionalized selenium nanoparticles for enhanced anti-hepatocarcinoma activity in vitro. *Nanoscale Research Letters*.

[12] Gour A., Jain N. K. (2019). Advances in green synthesis of nanoparticles. *Artificial Cells, Nanomedicine, and Biotechnology*.

[13] Yazdi M. T., Ghasemi Y., Ghasemian A. (2005). Bioconversion of hydrocortisone by cyanobacterium Fischerella ambigua PTCC 1635. *World Journal of Microbiology and Biotechnology*.

[14] Alagesan V., Venugopal S. (2019). Green synthesis of selenium nanoparticle using leaves extract of Withania somnifera and its biological applications and photocatalytic activities. *BioNano Science*.

[15] Gunti L., Dass R. S., Kalagatur N. K. (2019). Phytofabrication of selenium nanoparticles from Emblica officinalis fruit extract and exploring its biopotential applications: antioxidant, antimicrobial, and biocompatibility. *Frontiers in Microbiology*.

[16] Ramamurthy C., Sampath K. S., Arunkumar P. (2013). Green synthesis and characterization of selenium nanoparticles and its augmented cytotoxicity with doxorubicin on cancer cells. *Bioprocess and Biosystems Engineering*.

[17] Oremland R. S., Herbel M. J., Blum J. S. (2004). Structural and spectral features of selenium nanospheres produced by Se-respiring bacteria. *Applied and Environmental Microbiology*.

[18] Sharma G., Sharma A., Bhavesh R. (2014). Biomolecule-mediated synthesis of selenium nanoparticles using dried Vitis vinifera (raisin) extract. *Molecules*.

[19] Srivastava N., Mukhopadhyay M. (2013). Biosynthesis and structural characterization of selenium nanoparticles mediated by Zooglea ramigera. *Powder Technology*.

[20] Kalabegishvili T., Murusidze I., Kirkesali E. (2013). Gold and silver nanoparticles in Spirulina platensis biomass for medical application. *Ecological Chemistry and Engineering*.

[21] Chen T., Yang F., Wong K. H. (2014). Purification and in vitro antioxidant activities of tellurium-containing phycobiliproteins from tellurium-enriched Spirulina platensis. *Drug Design, Development and Therapy*.

[22] Uzair B., Liaqat A., Iqbal H. (2020). Green and cost-effective synthesis of metallic nanoparticles by algae: safe methods for translational medicine. *Bioengineering*.

[23] Moein M., Moein S., Fard T. B., Sabahi Z. (2017). Scavenging evaluation of different free radicals by three species of Ziziphus and their fractions. *Iranian Journal of Science and Technology, Transactions A: Science*.

[24] Sabahi Z., Zarshenas M. M., Farmani F., Faridi P., Moein S., Moein M. (2013). Essential oil composition and in vitro antioxidant activity of ethanolic extract of Thymus daenensis Celak from Iran. *Global Journal of Pharmacology*.

[25] Li Y., Li X., Wong Y. S. (2011). The reversal of cisplatin-induced nephrotoxicity by selenium nanoparticles functionalized with 11-mercapto-1-undecanol by inhibition of ROS-mediated apoptosis. *Biomaterials*.

[26] Klonowska A., Heulin T., Vermeglio A. (2005). Selenite and tellurite reduction by Shewanella oneidensis. *Applied and Environmental Microbiology*.

[27] Garbisu C., Ishii T., Leighton T., Buchanan B. B. (1996). Bacterial reduction of selenite to elemental selenium. *Chemical Geology*.

[28] Torres S. K., Campos V. L., León C. G. (2012). Biosynthesis of selenium nanoparticles by Pantoea agglomerans and their antioxidant activity. *Journal of Nanoparticle Research*.

[29] Ahmadi F., Bahmyari M., Akbarizadeh A., Alipour S. (2019). Doxorubicin-verapamil dual loaded PLGA nanoparticles for overcoming P-glycoprotein mediated resistance in cancer: effect of verapamil concentration. *Journal of Drug Delivery Science and Technology*.

[30] Cojocaru A., Sin I., Agapescu C., Cotarta A., Visan T. (2016). Electrode processes and SEM/EDX analysis of selenium films electrodeposited from ionic liquids based on choline chloride. *Chalcogenide Letters*.

[31] Çelekli A., Yavuzatmaca M., Bozkurt H. (2010). An eco-friendly process: predictive modelling of copper adsorption from aqueous solution on Spirulina platensis. *Journal of Hazardous Materials*.

[32] Yang F., Tang Q., Zhong X. (2012). Surface decoration by Spirulina polysaccharide enhances the cellular uptake and anticancer efficacy of selenium nanoparticles. *International Journal of Nanomedicine*.

[33] Kretzschmar J., Jordan N., Brendler E. (2015). Spectroscopic evidence for selenium(iv) dimerization in aqueous solution. *Dalton Transactions*.

[34] Chen Y.-W., Li L., D’Ulivo A., Belzile N. (2006). Extraction and determination of elemental selenium in sediments--a comparative study. *Analytica Chimica Acta*.

[35] Coccia F., Tonucci L., Bosco D., Bressan M., d'Alessandro N. (2012). One-pot synthesis of lignin-stabilised platinum and palladium nanoparticles and their catalytic behaviour in oxidation and reduction reactions. *Green Chemistry*.

[36] Mellinas C., Jiménez A., Garrigós M. D. C. (2019). Microwave-assisted green synthesis and antioxidant activity of selenium nanoparticles using Theobroma cacao L. bean shell extract. *Molecules*.

[37] Soumya R. S., Vineetha V. P., Reshma P. L., Raghu K. G. (2013). Preparation and characterization of selenium incorporated guar gum nanoparticle and its interaction with H9c2 cells. *PLoS One*.

[38] Chen Z., Shen Y., Xie A., Zhu J., Wu Z., Huang F. (2009). L-cysteine-assisted controlled synthesis of selenium nanospheres and nanorods. *Crystal Growth & Design*.

[39] Dehshahri A., Alhashemi S. H., Jamshidzadeh A. (2013). Comparison of the effectiveness of polyethylenimine, polyamidoamine and chitosan in transferring plasmid encoding interleukin-12 gene into hepatocytes. *Macromolecular Research*.

[40] Sabahi Z., Farmani F., Soltani F., Moein M. (2018). DNA protection, antioxidant and xanthine oxidase inhibition activities of polyphenol-enriched fraction of Berberis integerrima Bunge fruits. *Iranian Journal of Basic Medical Sciences*.

[41] Malhotra S., Welling M. N., Mantri S. B., Desai K. (2016). In vitro and in vivo antioxidant, cytotoxic, and anti-chronic inflammatory arthritic effect of selenium nanoparticles. *Journal of Biomedical Materials Research. Part B, Applied Biomaterials*.

